# Risk factors for SARS-CoV-2 pneumonia among renal transplant recipients in Beijing Omicron wave

**DOI:** 10.1128/spectrum.03005-23

**Published:** 2024-01-17

**Authors:** Guangping Li, Jiangtao Wu, Ying Huang, Qi Wang, Tianying Xing, Tongwen Ou

**Affiliations:** 1 Department of Urology, Xuanwu Hospital Capital Medical University, Beijing, China; Uniwersytet Medyczny w Bialymstoku, Bialystok, Poland

**Keywords:** SARS-CoV-2, Omicron, renal transplant recipients, risk factor

## Abstract

The novel coronavirus disease-19 had become an unprecedented global health emergency, quickly expanding worldwide. Omicron (B.1.1.529), as a novel variant of severe acute respiratory syndrome coronavirus 2 (SARS-CoV-2), was initially identified in South Africa and Botswana. Renal transplant recipients (RTRs) are a special group and are more vulnerable to viral pneumonia. Thus, this study aimed to assess the incidence and risk factors of SARS-CoV-2 pneumonia that occurred in RTRs with Omicron infection. This single-center case-control study enrolled the RTRs who were diagnosed with SARS-CoV-2 infection by the SARS-CoV-2 nucleic acid test, which were divided into two groups according to the imaging features of SARS-CoV-2 pneumonia. The parameters were collected by questionnaires and analyzed using Statistical Product and Service Solutions. A total of 313 RTRs completed the questionnaires, and 131 were enrolled in this study with a mean age of 42.66 years. The incidence of SARS-CoV-2 pneumonia among the enrolled participants was 76.3%. The first symptoms included fever (89.3%), cough (93.1%), and expectoration (81.7%). From the comparison, the parameters such as age, gender, body mass index, lymphocyte count, and the percent of neutrophils and the basic serum creatinine before SARS-CoV-2 infection were significantly different between the two groups (*P* < 0.05). In multivariate analysis, age and the basic serum creatinine were independent risk factors for developing SARS-CoV-2 pneumonia (*P* < 0.05). Older RTRs with a high level of serum creatinine before SARS-CoV-2 infection were more at risk of developing SARS-CoV-2 pneumonia. More randomized controlled studies are needed.

**IMPORTANCE:**

This study aimed to assess the incidence and the risk factors of severe acute respiratory syndrome coronavirus 2 (SARS-CoV-2) pneumonia that occurred in renal transplant recipients (RTRs) with Omicron infection. In conclusion, older RTRs with a high level of serum creatinine before SARS-CoV-2 infection were more at risk of developing SARS-CoV-2 pneumonia and should be timely treated, in case of severe pneumonia.

## INTRODUCTION

The novel coronavirus disease (COVID-19) had become an unprecedented global health emergency, quickly expanding worldwide. By 3 February 2023, according to the World Health Organization Coronavirus Dashboard, there had been 0.75 billion confirmed cases of COVID-19 caused by severe acute respiratory syndrome coronavirus 2 (SARS-CoV-2) and 6.8 million deaths globally (1). SARS-CoV-2, with a crown-like appearance, was embedded with homotrimer class I fusion S proteins on the external surface. Then SARS-CoV-2 could invade the host cells to bind to the receptor of angiotensin-converting enzyme 2 (ACE2) using the S protein. ACE2 was highly expressed in lung alveolar type II (AT2) cells. When the SARS-CoV-2 infected the lower airway, it could cause severe respiratory syndrome in humans (2).

Omicron (B.1.1.529), as a novel variant of SARS-CoV-2, was initially identified in South Africa and Botswana, named on 26 November 2021 based on the behaviors of mutations (3). Although the rapid spread of the Omicron variant caused global concern, growing evidence had shown that Omicron variant patients reported milder symptoms and better quality of life than the previous variants (4). Besides, the Omicron variant was more likely to cause upper respiratory infection, including sore throat, runny nose, and sneezing, but not lung infection. This infectious characteristic might be explained by the faster replication in the bronchi but less efficiently in the lung parenchyma (5).

Renal transplant recipients (RTRs) are a special group. Because of the history of chronic kidney disease and the use of immunosuppressants after transplantation, the symptoms, including fever, sore throat, and cough, in RTRs underwent SARS-CoV-2 infection seemed to be more severe, and they were more vulnerable to SARS-CoV-2 pneumonia, which deserved attention (6). Several studies had analyzed the risk factors of SARS-CoV-2 infection in RTRs, but no previous study was about SARS-CoV-2 pneumonia in this special group. So, the purpose of this study is to examine the incidence and risk factors of SARS-CoV-2 pneumonia in RTRs who were diagnosed with SARS-CoV-2 infection.

## MATERIALS AND METHODS

This was a single-center case-control study conducted in our renal transplantation center. RTRs who were diagnosed with SARS-CoV-2 infection by the SARS-CoV-2 nucleic acid test and underwent chest computed tomography (CT) scans were enrolled in this study between November 2022 and January 2023. All participants were divided into two groups according to the imaging features of SARS-CoV-2 pneumonia: pneumonia group and non-pneumonia group. Informed consent was signed by all enrolled participants.

The detection targets of the SARS-CoV-2 nucleic acid test were SARS-CoV-2 ORF1ab gene and N gene, with a lower detection limit of 500 copies/mL using the method of real-time fluorescence quantitative PCR. All enrolled participants underwent unenhanced chest CT scanning (MDCT-Sensation 64, Siemens Healthcare, Forchheim, Germany) by experienced radiologists after SARS-CoV-2 infection. The following typical CT manifestations were considered suggestive of SARS-CoV-2 pneumonia: ground-glass opacities, consolidation, reticular pattern, and crazy paving pattern (7).

We collected the relevant descriptive characteristics using questionnaires, including the basic information, renal transplant, and SARS-CoV-2 infection. Medical and personal histories were considered in this study, including diabetes mellitus, hypertension, and smoking history. We also recorded some laboratory indexes that reflected the level of immunity and the severity of infection, including tacrolimus (TAC), white blood cell (WBC), lymphocyte (LYMPH), red blood cell (RBC), hemoglobin (HGB), the percent of neutrophil (NEUT), platelets (PLT), alanine aminotransferase (ALT), aspartate aminotransferase (AST), lactic dehydrogenase (LDH), serum creatinine (Cr), albumin (A), globulin (G), and A/G. All indexes were obtained from the results of the latest tests of plasma tacrolimus concentration, blood routine test, and laboratory biochemistry before SARS-CoV-2 infection.

Statistical analysis was performed using Statistical Product and Service Solutions version 23.0 and GraphPad Prism version 8.4.3. Shapiro-Wilk test was used to evaluate the distribution of continuous variables. If the variable fitted the normal distribution, the parameter was compared with an independent-sample *t*-test between two groups and described as mean ± standard deviation. If not, the parameter was analyzed with a rank-sum test and described as median and interquartile ranges. Simultaneously, categorical variables were evaluated using a chi-square test between the two groups. Then the multivariate analysis was performed in the significantly different parameters using logistic regression. A receiver operating characteristic (ROC) curve was used to analyze the sensitivity, specificity, and cut-off value of the independent risk factors. *P* < 0.05 was considered statistically significant.

## RESULTS

**Fig 1:**
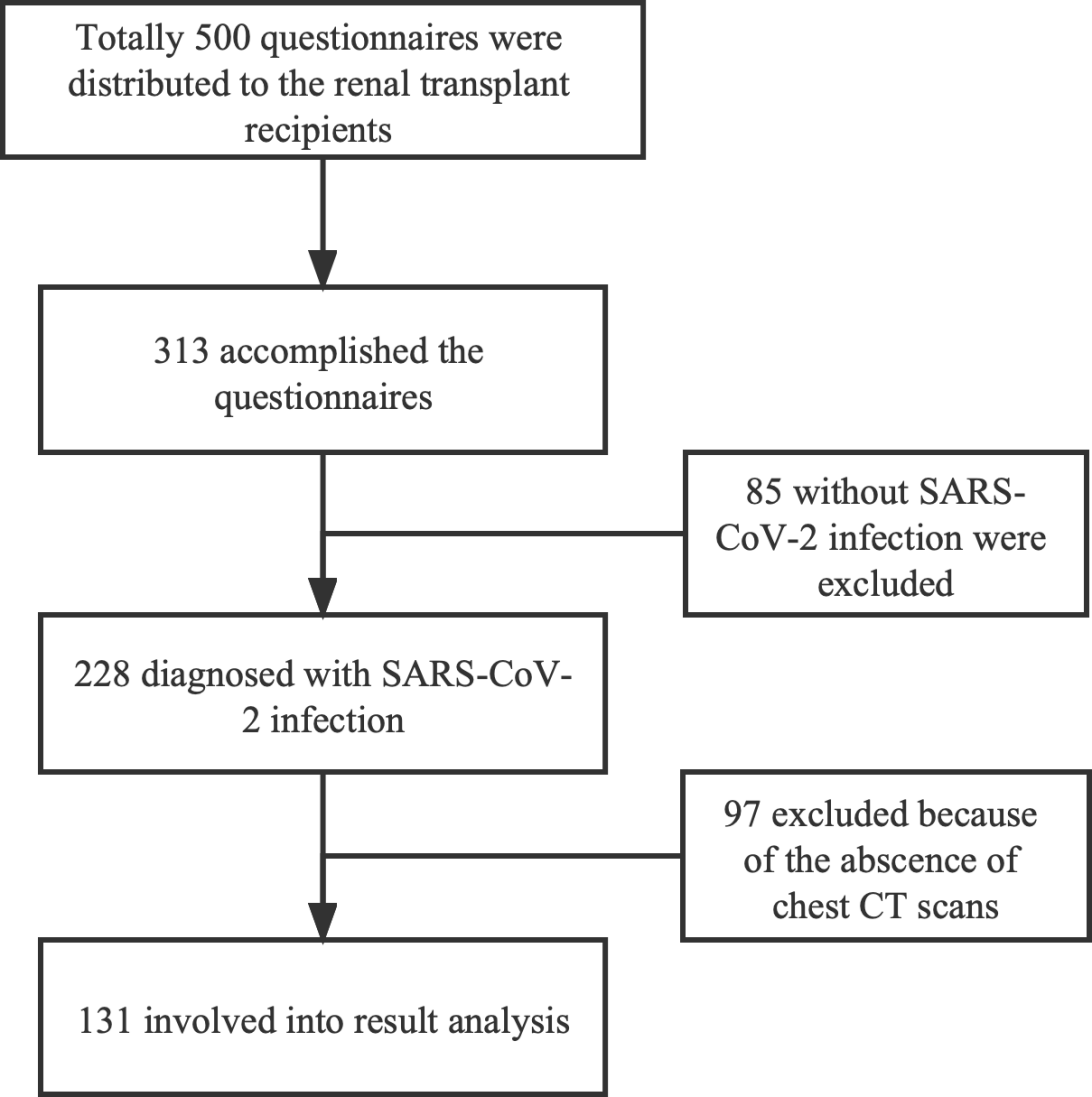
The process diagram of enrolling participants.

A total of 313 RTRs completed the questionnaires, and 228 recipients were diagnosed with SARS-CoV-2 infection by SARS-CoV-2 nucleic acid test. Among them, 97 recipients were excluded because of the absence of the chest CT scans. There were 131 RTRs involved in the result analysis. The process diagram is illustrated in Fig. 1, and the basic characteristics of all participants are listed in Table 1. The results showed that the mean age of RTRs with SARS-CoV-2 infection was 42.66 (±9.45) years and 40 recipients were female (30.5%). The incidence of SARS-CoV-2 pneumonia among the enrolled participants was 76.3%. Vaccinated RTRs were only in the minority (19/131, 14.5%), with one dose accounting for 3.1%, two doses 5.3%, and three doses 5.3%. The infectious symptoms of SARS-CoV-2 included cough (93.1%), fever (89.3%), expectoration (81.7%), chest stuffiness (54.2%), dizziness (35.9%), sore throat (28.2%), fatigue (22.1%), body aches (17.6%), urination problems (8.4%), diarrhea (6.9%), and tachycardia (4.6%).

**TABLE 1:** Basic characteristics of participants^*a*^

Characteristics	*N* = 131
Age (years), mean ± SD	42.66 ± 9.45
Gender, *N* (%)	
Female	40 (30.5)
BMI (kg/m^2^), mean ± SD	22.55 ± 4.10
Hemodialysis before transplantation, *N* (%)	93 (71.0)
Duration of RRT (months), median (IQR)	12 (3, 24)
Post-transplantation time (months), median (IQR)	11 (3, 20)
SARS-CoV-2 pneumonia, *N* (%)	100 (76.3)
Vaccination, *N* (%)	19 (14.5)
None	113 (86.3)
One dose	4 (3.1)
Two doses	7 (5.3)
Three doses	7 (5.3)
Symptoms, *N* (%)	
Cough	122 (93.1)
Fever	117 (89.3)
Expectoration	107 (81.7)
Chest stuffiness	71 (54.2)
Dizziness	47 (35.9)
Sore throat	37 (28.2)
Fatigue	29 (22.1)
Body aches	23 (17.6)
Urination problems	11 (8.4)
Diarrhea	9 (6.9)
Tachycardia	6 (4.6)
Viral pneumonia on chest CT, *N* (%)	100 (76.3)
Medical history, *N* (%)	
Diabetes mellitus	19 (14.5)
Hypertension	124 (94.7)
Smoking history, *N* (%)	29 (22.10)
Laboratory examinations	
TAC, ng/mL	6.10 (5.20, 7.00)
WBC, ×10^9^/L	7.57 (5.65, 9.30)
LYMPH, *×*10^9^/L	1.81 ± 0.90
RBC, *×*10^9^/L	4.16 ± 0.81
HGB, g/L	122.00 ± 21.55
NEUT, %	66.30 ± 11.95
PLT, ×10^9^/L	210 (183, 252)
ALT, IU/L	15 (11, 21)
AST, IU/L	17 (14, 21)
LDH*,* IU/L	209 (179.50, 269.00)
Cr, μmol/L	124 (101, 152)
A, g/L	44.05 (39.72, 46.67)
G, g/L	23.42 ± 3.50
A/G	1.86 ± 0.36

^*a*^ SD=standard deviation, BMI=body mass index, RRT=renal replacement therapy, IQR=inter quartile range, TAC=tacrolimus, WBC=white blood cell, LYMPH=lymphocyte, RBC=red blood cell, HGB=hemoglobin, NEUT=neutrophil, PLT=platelets, ALT=alanine aminotransferase, AST=aspartate aminotransferase, LDH=lactic dehydrogenase, Cr=creatinine, A=albumin, G=globulin.

According to the imaging features of SARS-CoV-2 pneumonia, 131 participants were divided into two groups, 100 participants in the pneumonia group and 31 in the non-pneumonia group. The results indicated that the age (43.88 vs 38.71 years, *P* = 0.007), BMI (22.88 vs 20.73 kg/m^2^, *P* = 0.049), NEUT (68% vs 61.50%, *P* = 0.028), and basic Cr before SARS-CoV-2 infection (129.00 vs 109.00 µmol/L, *P* = 0.013) were significantly higher in the pneumonia group than those parameters in the non-pneumonia group, while the level of LYMPH was significantly lower (1.64 vs 1.97 × 10^9^ /L, *P* = 0.049). Males with RTRs were more at risk of developing SARS-CoV-2 pneumonia (74% vs 54.8%, *P* = 0.043). There were no significant differences in the duration of renal replacement therapy before renal transplantation, post-transplantation time, vaccination, medical history of diabetes mellitus and hypertension, smoking history, TAC, WBC, RBC, HGB, PLT, ALT, AST, LDH, A, G, and A/G between the two groups (*P* > 0.05) (Table 2).

**TABLE 2:** Comparison between the pneumonia group and the non-pneumonia group^*a*^

Characteristics	Pneumonia group *N* = 100	Non-pneumonia group *N* = 31	*P*-value
Age (years), mean ± SD	43.88 ± 9.55	38.71 ± 8.09	0.007
Gender, *N* (%)			
Female	26 (26.0)	14 (45.2)	0.043
BMI (kg/m^2^), median (IQR)	22.88 (20.35, 25.65)	20.73 (17.51, 23.49)	0.012
Duration of RRT (months), median (IQR)	12.00 (4.00, 24.00)	8.50 (3.00, 15.75)	0.218
Post-transplantation time (months), median (IQR)	10.50 (3.00,19.75)	12.00 (7.00, 23.00)	0.066
Vaccination, *N* (%)	15 (15)	4 (12.9)	0.772
Medical history, *N* (%)			
Diabetes mellitus	17 (17.0)	2 (6.5)	0.150
Hypertension	95 (95)	29 (93.5)	0.577
Smoking history, *N* (%)	24 (24)	5 (16.1)	0.356
Laboratory examinations			
TAC, ng/mL	6.05 (5.20, 6.88)	6.20 (5.20, 7.40)	0.445
WBC, *×*10^9^/L	7.64 (6.12, 9.30)	7.07 (5.48, 9.30)	0.528
LYMPH, *×*10^9^/L	1.64 (1.14, 2.31)	1.97 (1.44, 2.53)	*0.049*
RBC, *×*10^9^/L	4.15 ± 0.85	4.20 ± 0.67	0.747
HGB, g/L	122.00 ± 21.39	122.00 ± 22.41	0.959
NEUT, *%*	68.00 (59.30, 75.03)	61.50 (55.60, 68.10)	*0.014*
PLT, *×*10^9^/L	207.50 (182.50. 247.75)	219.00 (183.00, 219.00)	0.172
ALT, IU/L	15 (12, 21)	13 (9, 21)	0.128
AST, IU/L	17.00 (14.00, 20.75)	17.00 (15.00, 21.00)	0.830
LDH, IU/L	213.0 (185.0, 271.5)	197.0 (169.0, 254.0)	0.124
Cr, *μ*mol/L	129.00 (106.50, 160.75)	109.00 (92.00, 138.00)	*0.013*
*A,* g/L	42.55 ± 5.30	48.83 ± 4.86	0.232
G, g/L	23.65 ± 4.50	23.43 ± 3.56	0.807
A/G	1.85 ± 0.35	1.88 ± 0.40	0.707

^*a*^ SD=standard deviation, BMI=body mass index, RRT=renal replacement therapy, IQR=inter quartile range, TAC=tacrolimus, WBC=white blood cell, LYMPH=lymphocyte, RBC=red blood cell, HGB=hemoglobin, NEUT=neutrophil, PLT=platelets, ALT=alanine aminotransferase, AST=aspartate aminotransferase, LDH=lactic dehydrogenase, Cr=creatinine, A=albumin, G=globulin.

**Fig 2:**
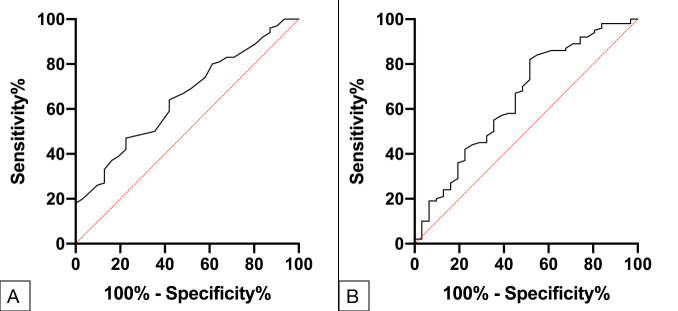
Forest plot of significant risk factors.

**Fig 3:**
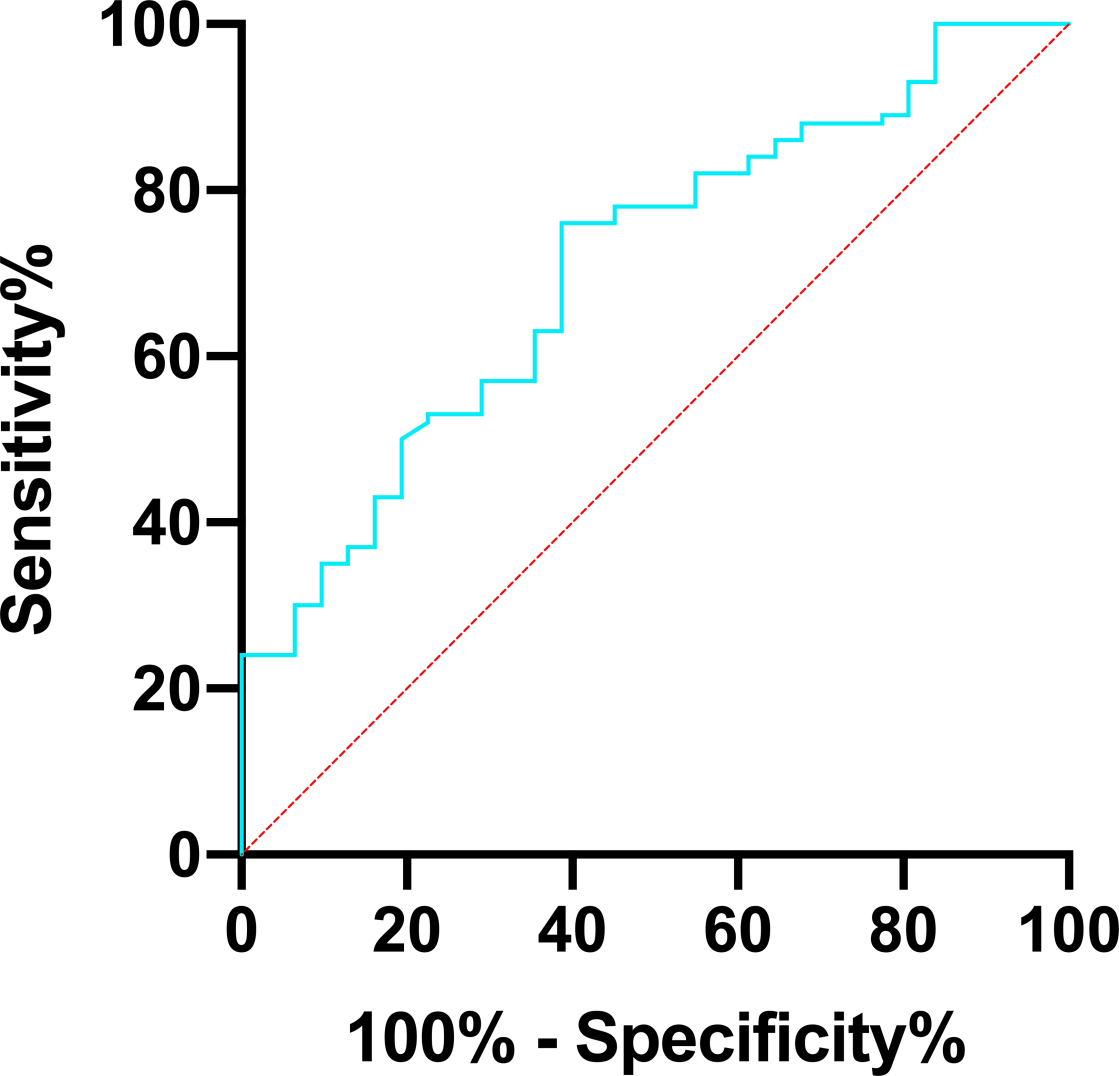
Receiver operating characteristic curve analysis of age (**A**) and creatinine (**B**).

**Fig 4:**
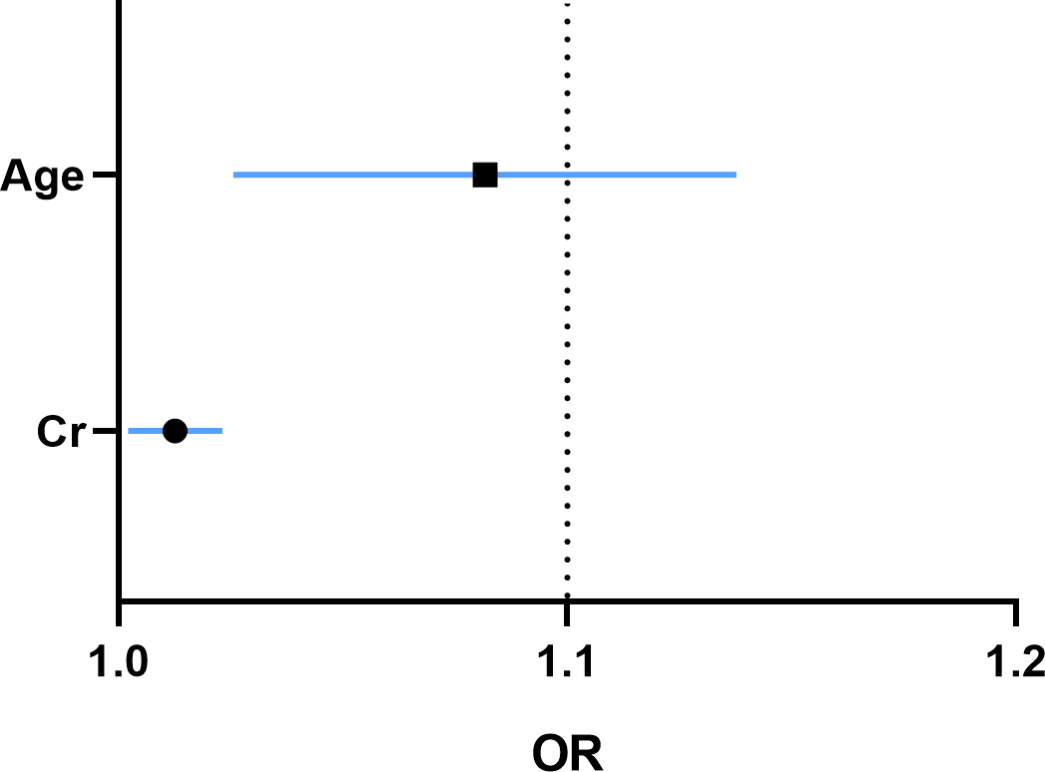
Receiver operating characteristic curve analysis of combined age and creatinine.

Furthermore, the multivariate logistic regression analysis showed that age and the level of basic serum Cr prior to the SARS-CoV-2 infection were independent risk factors of developing SARS-CoV-2 pneumonia in RTRs (Fig. 2; Table 3). In the ROC curve analysis, the parameter age had a high specificity (77.42%, *P* = 0.013) and basic Cr had a high sensitivity (82%, *P* = 0.013) to the occurrence of SARS-CoV-2 pneumonia (Fig. 3). By using the logistic regression, we combined the parameters age and basic Cr as the final regression model. The sensitivity and specificity of the model were 76% and 61.29%, respectively, with an AUC of 0.707 (*P* = 0.001) (Fig. 4; Table 4).

**TABLE 3:** Multivariable logistical regression and forest plot of significant risk factors^*a*^

Characteristics	B	OR	95% CI	*P*-value
Age (years)	0.078	1.081	1.026–1.138	0.003
Cr, μmol/L	0.013	1.013	1.002–1.023	0.018
Gender	-	-	-	0.184
BMI (kg/m^2^)	-	-	-	0.183
LYMPH, *×*10^9^/L	-	-	-	0.198
NEUT, %	-	-	-	0.165

^*a*^ OR=odds ratio, CI=confidence interval, Cr=creatinine, BMI=body mass index, LYMPH=lymphocyte, NEUT=neutrophil.

**TABLE 4:** ROC curve data of significant risk factors^*a*^

Characteristics	AUC	Cut-off value	Sensitivity %	Specificity %	95% CI	*P*-value
Age (years)	0.648	43.5	47	77.4	0.543–0.753	0.013
Cr, μmol/L	0.647	102	82	48.4	0.531–0.764	0.013
Age +Cr	0.707	115	76	61.3	0.608–0.807	0.001

^*a*^ ROC=receiver operating characteristic, CI=confidence interval, Cr=creatinine.

## DISCUSSION

During the Beijing Omicron wave, our center showed that 72.8% of RTRs were diagnosed with SARS-CoV-2 infection by the SARS-CoV-2 nucleic acid test. The most common symptoms were cough (93.1%), fever (89.3%), and expectoration (81.7%). Other symptoms included chest stuffiness (54.2%), dizziness (35.9%), sore throat (28.2%), fatigue (22.1%), body aches (17.6%), urination problems (8.4%), diarrhea (6.9%), and tachycardia (4.6%). According to the imaging characteristics, 76.3% of the patients developed SARS-CoV-2 pneumonia, higher than the number of previous COVID-19 infections that occurred in India (50%) (8). Only one previous study in Pakistan identified the possible risk factors associated with COVID-19 infection in renal transplant recipients (9), but no study in the literature assessed the risk factors of SARS-CoV-2 pneumonia that occurred in RTRs with SARS-CoV-2 infection.

Many pulmonary diseases, including chronic obstructive pulmonary disease (COPD), viral pneumonia, and pulmonary fibrosis, were caused by uncontrolled inflammation. In patients with COVID-19 infection, inflammation also played an important role in the development and progression of SARS-CoV-2 pneumonia. According to the results from several studies, SARS-CoV-2 could cause injury by host immune dysregulation and hyperinflammation (10). Further injuries, such as diffused alveolar damage and apoptotic epithelial cells induced by inflammation in the lungs, occurred resulting in high rates of hospitalizations and mortality (11).

The results from this study showed that an age >43.5 years, male, lower LYMPH, higher BMI, the percent of NEUT, and basic Cr before SARS-CoV-2 infection increased the risk of developing SARS-CoV-2 pneumonia in RTRs. Previous observations suggested that LYMPH and NEUT might be correlated with infection severity. Compared to the mild COVID-19 cases, the severe cases tended to have lower LYMPH and higher leukocyte counts (12). This was probably because the lower LYMPH indicated lower NK, T, and B cells, which were related to in-hospital death and severe illness (13). Furthermore, there was evidence that lymphopenia was correlated with palpitations and chest tightness on exertion, inducing persistent symptoms in COVID-19 survivors (14). That might be explained by T-cell dysfunction and the presence of auto-antibodies, which was correlated with the shedding of SARS-CoV-2 (15). NEUT elevation might result from the dysregulated expression of inflammatory cytokines and the upregulation of genes involved in the lymphocyte cell death pathway (16). Besides, a cohort study found that increased baseline serum Cr and in-hospital death were closely linked in RTRs, but it remained uncertain whether it was due to immunosuppression and the increased rate of renal dysfunction (17). Several recent studies found similar risk factors in SARS-CoV-2 infection. From the results of the European Renal Association COVID-19 Database (ERACODA) database, most of the enrolled 305 RTRs with COVID-19 were males with a mean age of 60  ±  13  years (18). The single center also showed that COVID-19 infection in this RTR group affected men more than women (19). In our study, the two groups in the gender category had statistically comparative differences, and there was a preponderance of males in both groups.

Chronic disease usually affects the obese population, as well as the young and the elderly. A cross-sectional study found that people with obesity were more likely to test positive for SARS-CoV-2 than those with normal weight (20). A meta-analysis study analyzed 30 related studies and enrolled 45,650 participants with COVID-19 infection to assess the risk of BMI-defined obesity. The results concluded that obesity, especially visceral adiposity, could increase hospitalization, intensive care unit admission, and death ratio (21). In our study, BMI in RTRs with SARS-CoV-2 pneumonia was significantly higher than those without SARS-CoV-2 pneumonia (*P* = 0.012). A previous study had identified that compared with nonsmokers, the pulmonary ACE2 gene expression was upregulated in ever-smokers, indicating an increased risk of viral binding and entry of SARS-CoV and SARS-CoV-2 in the lungs of smokers (22). However, in this study, there was no significant difference in the percentage of patients with smoking history between the pneumonia group and the non-pneumonia group (*P* = 0.356). It was perhaps because in 131 RTRs involved in this study, only 29 (22.10%) RTRs had smoking history. Thus, the characteristic of a smoking history was excluded from the multivariate logistical regression.

Furthermore, our study indicated two independent risk factors prior to the SARS-CoV-2 infection. Age and basic Cr levels played significant roles in the development of SARS-COV-2 pneumonia. Previous studies had described that age was a well-recognized risk factor for severe outcomes of COVID-19 infection. In England, over 90% of COVID-19 deaths occurred in patients over 60 years old (23). The results from OpenSAFELY also pointed to the strong association between increasing age and risk. The risk of death in patients over 80 years was 20 times higher than in those 50–59 years (24). From the results of the ERACODA collaboration, age was identified as the most important risk factor for mortality in RTRs. The mean age of the cohort was 60  ±  13  years, with 23.6% in-hospital mortality rate, higher than that of the general population (18).

Several studies compared the baseline serum creatinine between cadaveric and living RTRs with SARS-CoV-2 infection and found that cadaveric RTRs had significantly higher mean creatinine (8). The findings of the study in Pakistan also reported that a higher post-transplant serum creatinine (*P* = 0.019) was positively associated with COVID-19 infection (9). Compared to the general population, the creatinine level in RTRs was higher and more likely to develop acute kidney injury (*P* = 0.001) (25).

However, this study has several limitations. Bias might arise because this was a single-center study, and all enrolled RTRs were divided into two groups according to the imaging features of SARS-CoV-2 pneumonia, not randomly. Several potentially relevant characteristics, such as blood oxygen saturation, related CD (cluster of differentiation) molecules, and inflammatory indicators were not included in this study. Besides, the small sample size limited, to some extent, the generalization of the results, and more renal transplant centers and recipients needed to be enrolled to obtain further scientific analysis.

### Conclusion

Higher age, BMI, NEUT, basic Cr, and lower LYMPH were significantly associated with SARS-CoV-2 pneumonia. Multivariate regression analysis showed that age and the level of serum creatinine before SARS-CoV-2 infection were the independent risk factors, and strictly controlling the risk factors was beneficial for the prevention of SARS-CoV-2 pneumonia. However, considering the limitation of the sample size, more randomized controlled studies are needed.

## Data Availability

For compliance with the local laws and regulations, the sharing of data in a study involving human subjects is limited. The data that support the findings of this study are available upon reasonable request from the corresponding author, Tongwen Ou.
